# Solution Structure and Phylogenetics of Prod1, a Member of the Three-Finger Protein Superfamily Implicated in Salamander Limb Regeneration

**DOI:** 10.1371/journal.pone.0007123

**Published:** 2009-09-22

**Authors:** Acely Garza-Garcia, Richard Harris, Diego Esposito, Phillip B. Gates, Paul C. Driscoll

**Affiliations:** 1 Division of Molecular Structure, MRC National Institute for Medical Research, London, United Kingdom; 2 Institute of Structural and Molecular Biology, University College London, London, United Kingdom; Centre for Genomic Regulation (CRG), Universitat Pompeu Fabra, Spain

## Abstract

**Background:**

Following the amputation of a limb, newts and salamanders have the capability to regenerate the lost tissues via a complex process that takes place at the site of injury. Initially these cells undergo dedifferentiation to a state competent to regenerate the missing limb structures. Crucially, dedifferentiated cells have memory of their level of origin along the proximodistal (PD) axis of the limb, a property known as positional identity. *Notophthalmus viridescens* Prod1 is a cell-surface molecule of the three-finger protein (TFP) superfamily involved in the specification of newt limb PD identity. The TFP superfamily is a highly diverse group of metazoan proteins that includes snake venom toxins, mammalian transmembrane receptors and miscellaneous signaling molecules.

**Methodology/Principal Findings:**

With the aim of identifying potential orthologs of Prod1, we have solved its 3D structure and compared it to other known TFPs using phylogenetic techniques. The analysis shows that TFP 3D structures group in different categories according to function. Prod1 clusters with other cell surface protein TFP domains including the complement regulator CD59 and the C-terminal domain of urokinase-type plasminogen activator. To infer orthology, a structure-based multiple sequence alignment of representative TFP family members was built and analyzed by phylogenetic methods. Prod1 has been proposed to be the salamander CD59 but our analysis fails to support this association. Prod1 is not a good match for any of the TFP families present in mammals and this result was further supported by the identification of the putative orthologs of both CD59 and *N. viridescens* Prod1 in sequence data for the salamander *Ambystoma tigrinum*.

**Conclusions/Significance:**

The available data suggest that Prod1, and thereby its role in encoding PD identity, is restricted to salamanders. The lack of comparable limb-regenerative capability in other adult vertebrates could be correlated with the absence of the Prod1 gene.

## Introduction

Regeneration of damaged or missing body parts in adulthood is fairly widespread in invertebrate animals, but relatively rare in vertebrates. The most extensive regenerative ability among adult vertebrates is found among various species of salamanders, the urodele amphibians, which are able to replace a variety of structures including the limbs, tail, jaws and spinal cord. One important step in urodele regeneration appears to be the ability to create stem cell-like progenitor cells from already differentiated cells, a process known as dedifferentiation. The process of regeneration in urodele limbs has been particularly well-characterized: upon amputation, epidermal cells migrate to cover the wound, and subsequently cells under the epidermis revert to mesenchymal stem cells and form a mound at the end of the stump called a blastema [1], which then grows and differentiates to re-form the tissues. Importantly, the blastema gives rise only to that part of the limb distal to its level of origin; for example, a blastema formed after amputation at the level of the wrist leads to the regeneration of only the missing hand, whereas an entire arm is formed from a blastema that arises following amputation at the shoulder. Moreover, the blastema is an autonomous morphogenetic entity, as its ‘positional memory’ is conserved even when excised and grafted onto another site of the body [2]. Thus, a given blastema is distinguished at the cell and molecular level according to the site of its origin along the proximodistal (PD) axis (shoulder to fingertip). PD identity appears to be encoded, at least partly, by the expression of the 87-residue cell-surface protein Prod1, the cDNA of which was originally detected from a differential screen on retinoic acid treated limb bud blastemas of the Eastern Newt, *Notophthalmus viridescens* [3]. Phosphatidylinositol phospholipase C releases Prod1 from the cell surface, suggesting that it is bound to the membrane by a glycosylphosphatidylinositol (GPI) anchor. By sampling cells from the intact newt limb, the level of expression of Prod1 was found to correlate with the PD position, with higher levels at proximal positions. Retinoic acid, a modifier of blastema PD identity, increases the Prod1 expression level in distal blastemal cell [3], [4]. Moreover, antibodies raised against Prod1 alter the adhesivity of the blastemal cells [3], [5], and increasing the expression level of Prod1 proximalizes the cells in the regenerating limb [4], [6], [7].

It is not understood why most adult vertebrates are unable to regenerate. This could reflect either the absence of certain gene products, or alternatively the failure of those genes to act in an appropriate way following injury. The present-day understanding of regenerative mechanisms is only partial, but in general the genes that have been implicated belong to families that are widespread rather than being found only in taxa that are able to regenerate [7]. In this context, the identification of Prod1 as an important molecular component in urodele limb regeneration renders it imperative to understand its molecular phylogeny, and in particular to establish whether there are functional orthologs for Prod1 within other phylogenetic groups. Also, the discovery of Prod1 orthologs in model organisms such as the mouse or zebrafish would likely accelerate the elucidation of the functional mechanism of Prod1 action.

The Prod1 amino acid sequence codes for a secreted single-domain protein of the urokinase-type plasminogen activator receptor (uPAR)/Ly-6/CD59/snake toxin superfamily [8], also referred to as the three-finger protein (TFP) superfamily [9]. The TFP polypeptide fold is a multiple disulfide-bonded, mainly β-structure of 60–90 residues, and it is widely found in secreted soluble, GPI-anchored and single-pass transmembrane proteins. In the initial report describing the role of Prod1 in newt limb regeneration, the available sequence and structural information was interpreted to suggest that Prod1 is the newt ortholog of mammalian CD59, a protein with a well established role in the regulation of the complement system membrane attack complex [3]. Here we present the determination of the 3D solution structure of recombinant Prod1 using heteronuclear nuclear magnetic resonance (NMR) spectroscopy. In tandem we used sequence- and structure-based phylogenetic analysis to probe the relationship of Prod1 to known TFP superfamily proteins, and in particular to determine whether Prod1 is indeed newt CD59. The low sequence conservation of the TFP superfamily presents a challenge to the application of phylogenetic techniques, but the analysis of the available high resolution 3D structures for multiple TFP superfamily members, coupled with emerging urodele EST sequence information, leads to the unambiguous conclusion that Prod1 is not newt CD59. Moreover, the present data suggest that Prod1-like proteins are specific to newts and salamanders, and this finding has significance both for the interpretation of its role in PD identity and the phylogenetic restriction of limb regeneration.

## Results and Discussion

### Solution structure of Prod1 and comparison to the structures of other TFPs

**Figure 1:**
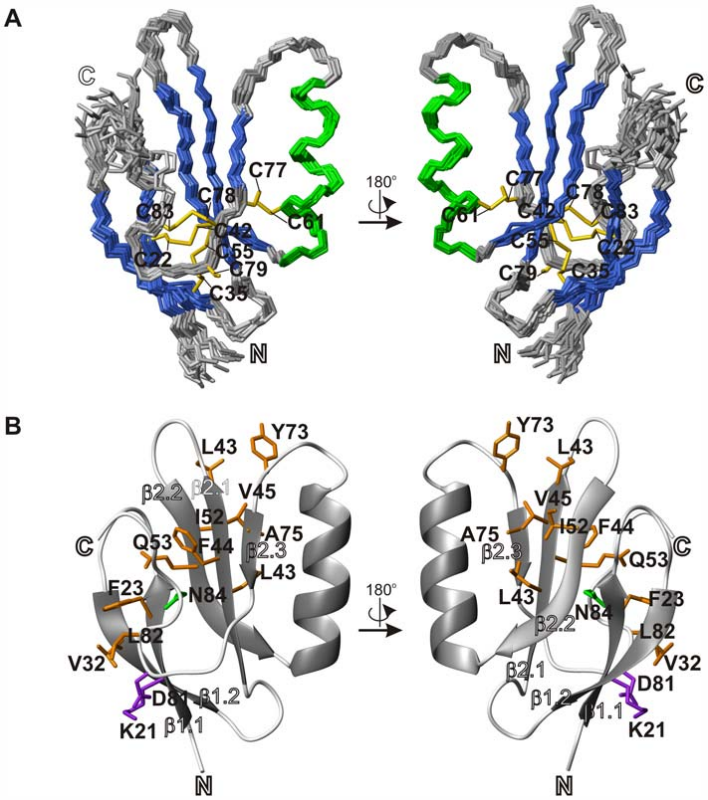
NMR solution structure of the TFP domain of Prod1. (a) Backbone atom traces of the ensemble of 20 lowest energy conformers. Helices are shown in green and β-strands in blue; the side chains of the cysteines are shown in yellow. (b) Ribbon representation of the lowest energy conformer of the ensemble. Conserved residues involved in hydrophobic interactions are shown in orange, and sidechains involved in hydrogen bonds in purple. The C-terminal Asn is shown in green.

The construct of Prod1, lacking the N-terminal signal sequence, was expressed in *Escherichia coli* as insoluble aggregates that were solubilized, purified, reduced and folded *in vitro*. *In vitro*-folded Prod1 was found to possess good solubility and stability and to yield high quality NMR spectra (Figure S1). The 3D solution structure was solved by standard restrained molecular dynamics-based simulated annealing calculations using inter-proton distance restraints derived from nuclear Overhauser effect (NOE) measurements and dihedral angle restraints obtained from backbone atom chemical shifts. Following iterative assignment of NOE cross peaks, the final round of calculations used an average of 3.6 long-range (i−j≥5) distance restraints per residue. The resulting 3D structure, represented by the 20-member conformer bundle depicted in Figure 1, is well defined by the experimental data, with measures of global coordinate precision and structural quality scores typical of NMR-derived solution structures of globular proteins (Table 1).

**Table 1:** Restraints and structural statistics of the Prod1 ensemble.

*Structural constraints*			
Inter-proton distance constraints			
	All	1122	
	Intra-residue	399	
	Sequential (|i−j| = 1)	258	
	Short (1<|*i−j*|>5)	169	
	Long (|i−j|≥5)	257	
Hydrogen bonds		18	
Dihedral angle constraints		88	
Disulphide bonds		4	
		Ensemble (n = 20)	Lowest energy
*Root mean square deviation from experimental data*			
Inter-proton distance restraints (Å)		0.032+/−0.001	0.03
Dihedral restraints (°)		0.89+/−0.07	0.84
*Deviation from idealised covalent geometry*			
Bonds (Å)		0.0051+/−0.0001	0.0053
Angles (°)		0.67+/−0.02	0.72
Improper dihedrals (°)		1.9+/−0.10	1.90
*Restraint violations*			
NOE violations >0.4 Å		1+/−0.50	1
Dihedral angle violations >4°		0.4+/−0.70	2
*Precision of the ensemble*			
Average pairwise root mean square deviation (Å) of:			
Backbone (bb) atoms (residues 20–88)		1.1+/−0.28	
Heavy atoms		1.77+/−0.28	
Regular secondary structure bb atoms (37 residues)		0.54+/−0.09	
Regular secondary structure heavy atoms		1.17+/−0.14	
*Ramachandran statistics (PROCHECK)*			
Most favoured region (%)		85.015+/−3.63	78.50
Additionally allowed regions (%)		13.99+/−3.42	18.50
Generously allowed regions (%)		0.98+/−1.03	3.10
Disallowed regions (%)		0.00	0
*WHAT IF quality scores*			
1st generation packing quality Z-score		−0.94+/−0.09	−1.07
2^nd^ generation packing quality Z-score		−2.58+/−0.53	−2.82
Backbone conformation Z-score		−0.68+/−0.56	−0.39
Ramachandran plot appearance Z-score		−3.72+/−0.35	−4.99
Chi-1 chi-2 rotamer normality Z-score		−4.19+/−0.67	−3.80
Improper dihedral distribution RMS Z-score		0.50+/−0.02	0.51

Prod1 has the slight concave disc shape characteristic of other TFP domains (Figure 1). The signature regular secondary structure features of the TFP fold are an N-terminal β-hairpin followed by a three-stranded antiparallel β-sheet. Loops β1/β2, β3/β4 and β4/β5 protrude from the core forming the tips of the “fingers”. For Prod1, the regular elements of secondary structure determined by the DSSP algorithm [10] are β1.1(21-25)−β1.2(32-36)−β2.1(42-47)−β2.2(52-57)−α–helix(59-70)−β2.3(76-79) where, for β*n.p*(*i−j*), *n* is the β-sheet number, *p* is the strand number within the β-sheet, and *i−j* the constituent residue range. In comparison to other TFPs, the 12-residue-long α-helix comprising the connection between strands β5 and β6 is the most distinctive feature of Prod1.

**Figure 2:**
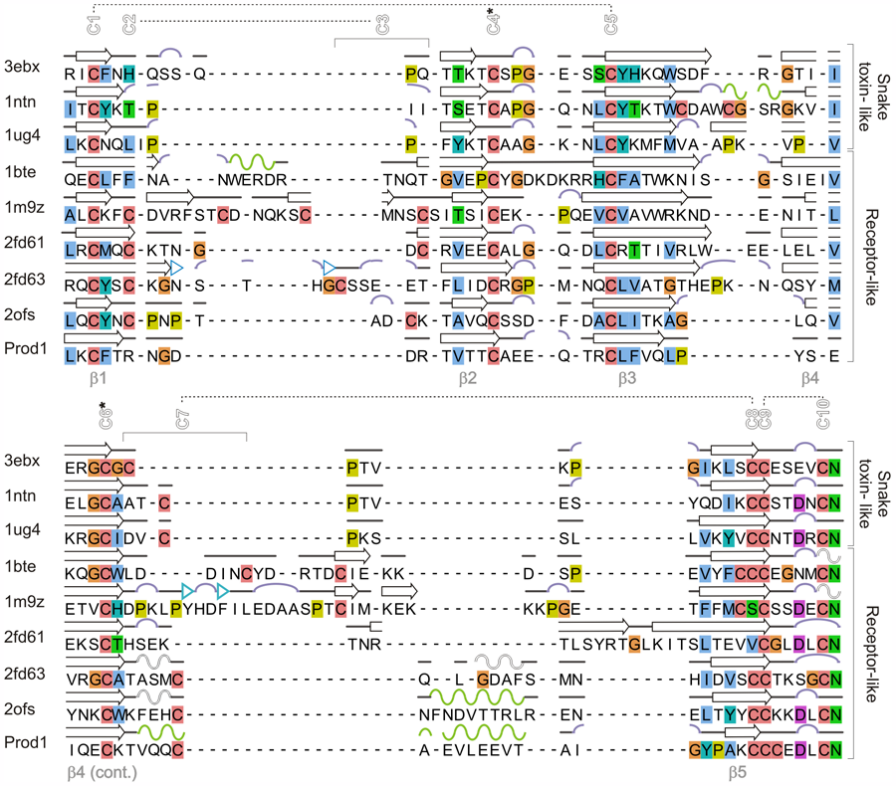
Structure-based multiple sequence alignment of selected TFP 3D structures highlighting the regular secondary structure features. Alpha-helices are shown in green, 3_10_- helices in white, β-strands in black, bends in purple and β-bridges in cyan. The canonical cysteines are numbered at the top and disulfide-bond connectivities are indicated by the dotted lines or by stars. Glycines are colored in orange, prolines in yellow and cysteines in pink; other positions are colored according to conservation of chemical properties: hydrophobic in blue, aromatic in cyan, polar negative in purple, polar positive in red, and polar neutral in green.

The canonical TFP domain has 10 disulfide-bonded cysteines arranged in the pattern C1-C5, C2-C3, C4-C6, C7-C8 and C9-C10 (in order of occurrence of the cysteines in the sequence; Figure 2). Some TFP domains lack one of these disulfides; the most widely absent is C2-C3, which is the only one that is not in the core of the TFP fold. Additional disulfides are also present in some TFP sub-groups [11]. Prod1 has nine cysteines with connectivity Cys22-Cys42 (corresponding to the generic C1-C5 bond), Cys35-Cys55 (C4-C6), Cys61-Cys77 (C7-C8) and Cys78-Cys83 (C9-C10). Prod1 purified as a monomer as assessed by analytical size exclusion chromatography. Thus Cys79 is not involved in an intermolecular disulfide bond. The canonical TFP C2-C3 bond is replaced in Prod1 by the charged residue pair Arg25 and Asp28, and side chain contacts between these residues may help to stabilize the β1/β2 loop.

Another highly conserved residue among TFP domain-containing proteins is an Asn residue at the C-terminus of the domain. In most TFP 3D structures, including Prod1, this Asn bridges the C-terminus to β-strands β1, β3 and β4 and interacts with the residue following the first canonical cysteine (denoted here C1+1; Phe23 in Prod1) and – using this same labeling of positions with respect to the canonical cysteines – with the residues at positions C5+1 (Leu43), C5+2 (Phe44), C6−2 (Gln53) and C6−1 (Glu54). Other conserved contacts, which in some TFP structures have a hydrophobic character and in others are charge-pair interactions, are between the side chains of residues at positions C1−1 and C10−2 (Lys21 and Asp81 in Prod1); C1+1 and C6−2 (Phe23 and Gln53) ; C1+1 and C10−1 (Phe23 and Leu82); C4−3 and C10−1 (Val32 and Leu82); C5+1 and C6+1 (Leu43 and Ile52); C5+1 and C8−2 (Leu43 and Ala75); C5+2 and C6−2 (Phe44 and Gln53); C5+3 and C8−2 (Val45 and Ala75); C5+3 and C8−4 (Val45 and Tyr73) (Figure 1). TFP domains typically lack a classical hydrophobic core, but possess a few partly-exposed hydrophobic side chain clusters. The only fully buried hydrophobic interaction in Prod1 is between Leu43 and Val45. Three partly-exposed hydrophobic residues (Phe23, Val32 and Leu82) bridge the N-terminal β-hairpin to the irregular C-terminus of the domain in an arrangement that is common in TFP structures. Additional hydrophobic interactions made by residues at positions C5+1 (Leu43 in Prod1), C5+5 (Leu47), C8−4 (Tyr73) and C8−2 (Ala75), are also well-conserved across the TFP superfamily.

### Relationship of Prod1 to other TFP superfamily structures

Establishing the relationship of Prod1 to other members of the TFP superfamily should aid the identification of orthologous molecules, and also point to residues that mediate intermolecular interactions and provide an insight into possible modes of action. Most often, molecular phylogeny is based upon the analysis of alignments of nucleotide or amino acid sequences. However, the construction of a well-supported protein sequence-based phylogenetic relationship of the TFP superfamily encounters particular challenges: (1) the multiple sequence alignment has many ambiguous regions due to the low overall sequence identity between the members (as low as 20%); and (2) the resolving power of the analysis is compromised by the short sequence length of the domain and the presence of sites that have accumulated multiple residue substitutions, insertions and deletions so as to obscure the phylogenetic signal, a phenomenon known as substitutional or mutational saturation [12]. An alternative strategy that could circumvent these obstacles is to calculate phylogeny using 3D structure information. As protein structures tend to evolve more slowly than their corresponding amino acid sequences [13], a phylogenetic analysis comparing 3D structures has the potential to detect similarities that have been lost at the sequence level. Our approach to the phylogenetic analysis of the TFP domain-containing proteins is founded on this concept.

In the Pfam database [14] the TFP superfamily (clan CL0117), is divided into five families according to similarity of protein architecture, function and sequence; three of the families have representative 3D structures in the PDB. Submission of the Prod1 3D structure to the protein structure comparison servers VAST [15], DALI [16] or FATCAT [17] generates significant hits to proteins within each of these three families, namely: a) the single-domain TFP toxins present in the venom of snakes from the Elapidae and Colubridae families (Pfam family PF00087); b) a heterogeneous family of proteins that includes CD59 and uPAR (PF00021); and c) the TFP domains present in the TGF-βreceptor family (in which the TFP ectodomain is found in combination with a cytoplasmic serine/threonine kinase domain; PF01064). This result could arise because the structure of Prod1 lies equidistant to the existing families, or simply because the scoring systems used by the similarity search algorithms are unable to differentiate between the families. In order to assess whether Prod1 could be assigned more specifically to any of these pre-established families, we calculated a structural distance-based phylogeny [18]–[20] by quantifying pairwise protein structure similarity between the available TFP 3D structures.

There is no unique method for measuring the degree of similarity between macromolecular structures. The traditional method of comparing structures as rigid bodies is usually not suitable for comparison between distantly-related structures, such as members of a superfamily, where relative reorientation of the conserved (often peripheral) secondary structure features is commonplace. Nevertheless, a variety of scoring methods that do allow for flexibility are available, some of which use the explicit atomic coordinates such as FATCAT [17], whilst others use alternative representations of the structures such as DALI [21] that compares the distance distributions between Cα atoms. We assessed FATCAT, DALI and other programs for their ability to cluster the structures of the TFP superfamily according to their function and their classification in the CATH database [22], and found the phenotypic plasticity method (PPM) to be the most successful. PPM attempts to measure the evolutionary cost of transforming one structure into another by means of residue substitutions, insertions and deletions, thus emulating amino acid sequence comparison using amino acid exchange matrices and gap penalties [23]. All of the methods tested are in agreement regarding the classification of Prod1 structure within the superfamily (Figures S3, S4, S5), but for simplicity, we opt to present here only the results using PPM scores.

**Figure 3:**
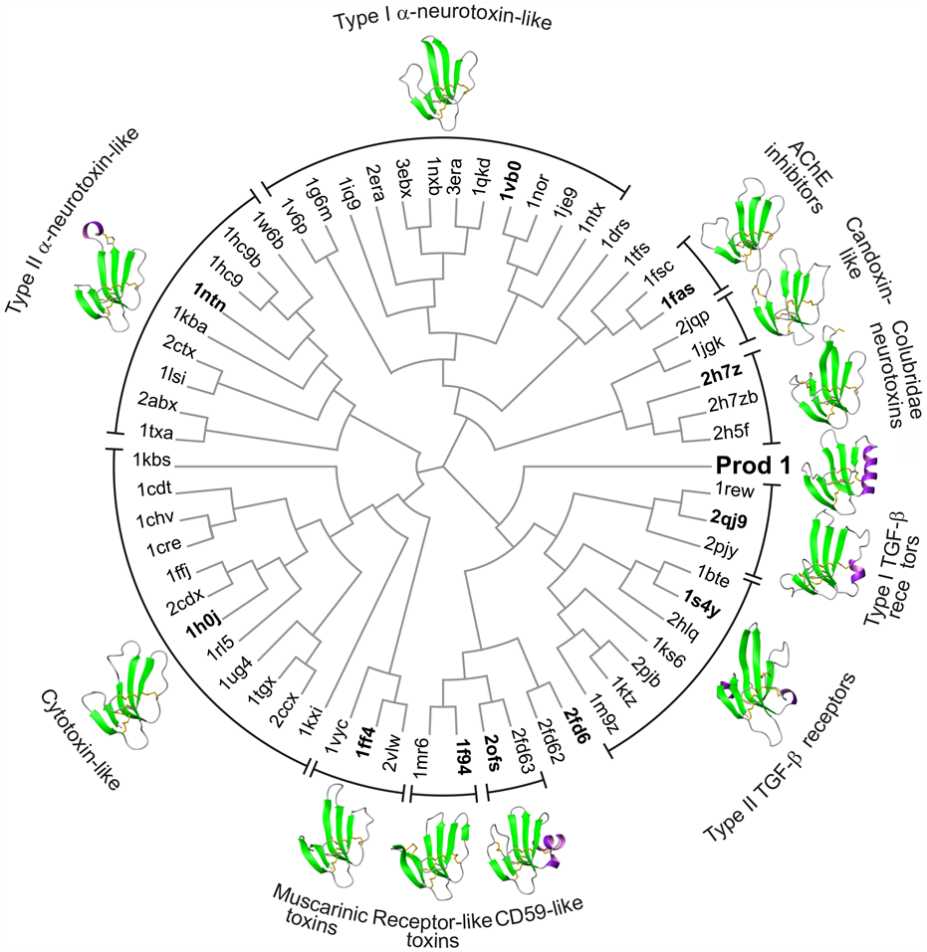
Structure-based phylogenetic tree of TFP domains. The tree was computed using BioNJ and a matrix of pairwise structure distances derived from phenotypic plasticity method (PPM) similarity scores. PDB codes in bold correspond to the structure adjacent structure shown in ribbon representation.

We located 61 TFP domain 3D structures with non-identical sequences from the PDB (see Materials and Methods) and used PPM to derive pairwise structural similarity scores, which were then converted to distance scores (Table S1). The distance matrix was then used to calculate a phylogenetic tree using the BIONJ algorithm [24] and a neighbornet network [25] (Figures 3 & S2). Phylogenetic networks allow for the representation of conflicting signals, uncertainty, ambiguity and non-tree-like evolutionary histories, and as such are emerging as a tool to assess uncertainty or conflict within the dataset prior to tree building [26], [27]. The fact that the clade composition of the neighbornet network for the TFP structures is in agreement with that of the BIONJ tree indicates that the clades in the tree are well supported by the raw data [28] .

The phylogenetic tree shows a primary split between snake venom and receptor-like proteins that can also be seen in the network. The snake-venom proteins are in turn clustered, mainly according to function, in seven groups: type I α-neurotoxins, type II α-neurotoxins, cytotoxins, neurotoxins from Colubridae snakes, acetylcholinesterase inhibitors, muscarinic neurotoxins and candoxin-like proteins. The receptor-like cluster contains four well-supported groups: the TFP domains of the type I receptors of TGF-β like proteins; the TFP domains of the type II receptors of TGF-β like proteins, the C-terminal domain of uPAR and CD59; and a clade of two snake venom proteins comprising the weak platelet aggregation inhibitor γ-bungarotoxin (1MR6) [29], and bucandin (1F94) [30] - a non-toxic protein of unknown function. It is interesting that the presence of the toxins in the receptor-like cluster could constitute evidence that the snake venom arsenal diversified from an ancestral harmless 10-cysteine protein, as has been suggested [11]. Most of the proteins in the receptor-like cluster possess the C2-C3 disulfide bond, but the position of C3 in the 3D structure is variable. These proteins also tend to have insertions between strands β1 and β2 and/or β4 and β5. The latter insertion often includes an extra segment of regular secondary structure, comprising either one or more short α-helices or a β-strand.

In our structure-based phylogenetic calculations, Prod1 was consistently located in the receptor-like cluster, although it was not found within any of the sub-groups we have just described. Among the cluster members, the most similar structure to Prod1 is CD59 with a PPM score of 62.98. However, this best match is not reciprocal as the PPM score between CD59 and the third domain of uPAR (2FD6-3) is 75.96; Prod1 ranks only fifth among the PPM scores for CD59. Similarity between Prod1 and CD59 is mainly due to the conformation and length of the insertion between β4 and β5, as they both have α-helices in this region and share the position of C7 in the structure. The absence of the C2-C3 bond in Prod1 does not result in significant structural differences compared to the corresponding region in CD59. Although a member of the extracellular receptor cluster by this analysis, Prod1 also shows substantial similarity to members of the muscarinic toxin group. Muscarinic toxin 2 (MT2) is the second best hit to Prod1 with a PPM score of 61.77; the resemblance of Prod1 to the muscarinic toxic group was observed using all of the assessed scoring systems.

There is one pair of known orthologous structures in the dataset: the TFP domain of the TGF-β receptor type 2 of human (1M9Z) and chicken (1KS6). The PPM score between these two 3D structures is 133.71, clearly much higher than the PPM score of 62.98 for Prod1 and CD59. This result indicates that the structure-based phylogenetic approach does not support that Prod1 and CD59 are orthologs. Having arrived at this conclusion using structural data, we resorted to the incorporation of the more abundant TFP sequence data into the analysis in order to gain further insight into the position of Prod1 within the TFP superfamily and to probe further for the existence of a mammalian ortholog.

### Prod1 sequence within the TFP superfamily

**Figure 4:**
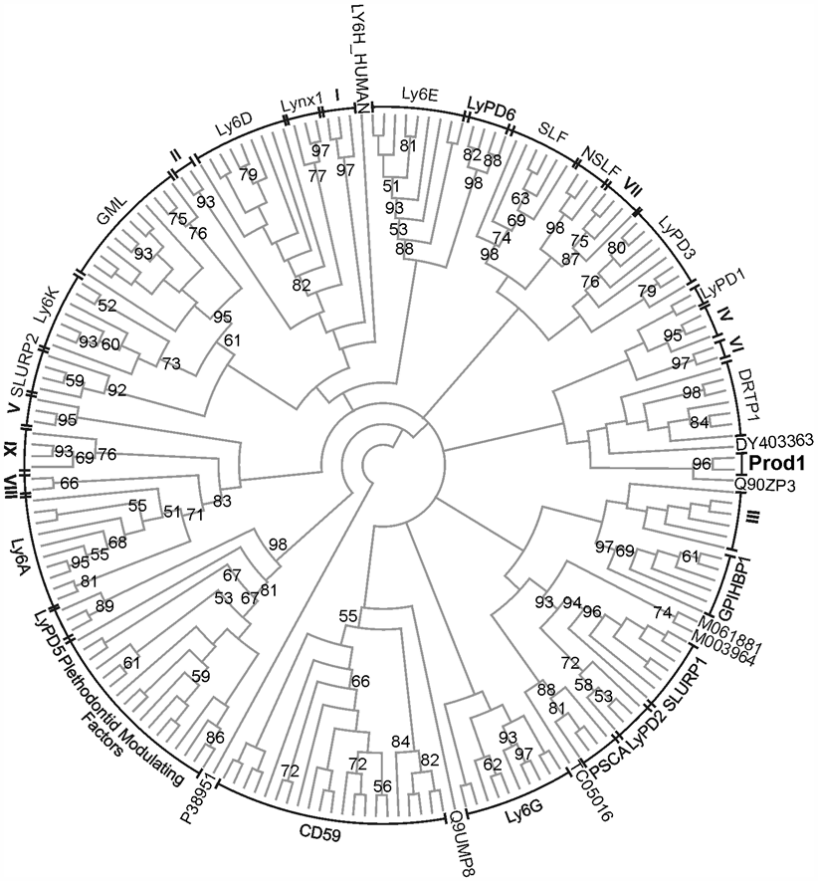
Majority-rule consensus phylogenetic tree of representative sequences of single-domain TFPs computed by maximum-likelihood using PhyML. Numbers refer to bootstrap confidence values; only those greater than 50% are shown. The sequence of the TFP domain of the Type II activin receptor was used to root the tree, but was removed from the figure. See Table S2 for the identity of the sequences in each grouping.

**Figure 5:**
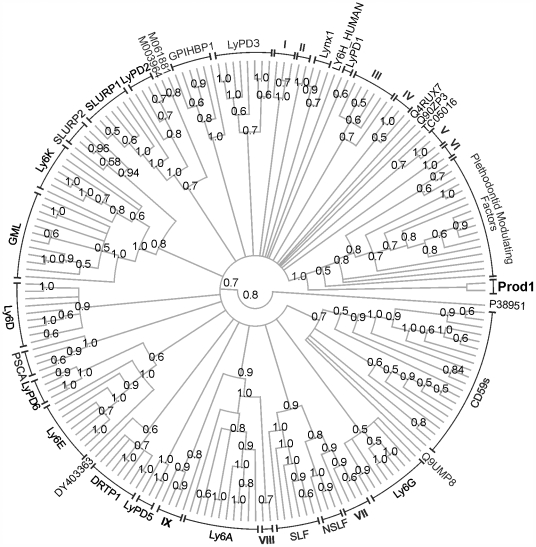
Majority-rule consensus phylogenetic tree of representative sequences of single-domain TFPs computed by Bayesian inference using MrBayes. Numbers indicate the clade credibility (posterior probability) values; only posterior probabilities grater than 0.50 are shown. The sequence of the TFP domain of the Type II activin receptor was used to root the tree, but was removed from the figure. See Table S3 for the identity of the sequences in each grouping.

To the best of our knowledge, a phylogenetic analysis of the TFP superfamily has not been reported previously. This is most likely due to the difficulty of comparing novel sequences to known TFP members given the low sequence similarity and wide functional diversity. Due to the short length and high variability of the sequences, the TFP superfamily is not an ideal candidate for sequence-based phylogenetic analyses, but the wealth of information that it could provide justifies the exercise, as long as measures are taken to minimize potential error and the relevant caveats are taken into account. As the reliability of the phylogeny output is strongly dependent upon the quality of the starting multiple sequence alignment, and the use of constraints derived from structural data has been shown to increase the accuracy of sequence alignments [31], we based our alignment of the TFP sequences using information from the multiple superposition of TFP 3D structures. Our approach was to first calculate a structure-based sequence alignment subset of the TFP structures in the receptor-like cluster, and then use this alignment to direct the alignment of a much larger representative set of TFP domain amino acid sequences (i.e. lacking 3D structures). Phylogenetic trees were then computed by maximum likelihood (Figure 4) and Bayesian analysis (Figure 5). Both methods clustered the sequences in 30 well-supported groups (Tables S2 and S3); all 30 groups are also present in the neighbornet network computed using a maximum-likelihood pairwise distance matrix (Figure S6). The phylogenetic classification of single-domain TFPs presented here serves to illuminate the patterns of relatedness among currently known single-domain TFP families and should provide a general basis for a more systematic categorization of novel TFP members.

A critically important outcome of the structure-based sequence phylogenetic reconstitution is the determination that, consistent with our analysis based upon TFP structures alone, Prod1 is not the ortholog of CD59. Thus CD59 clade was recovered in both the maximum likelihood and Bayesian phylogenies and the network shows that this grouping is well supported by the data. The CD59 clade contains sequences from fish, birds, reptiles, amphibians, marsupials and placentals, including the sequence TC00268 obtained from the tiger salamander, *Ambystoma tigrinum*. In common with *N. viridescens*, the newt in which Prod1 was found, A. tigrinum belongs to the suborder of the advanced salamanders (Salamandroidea). Human CD59 and TC00268 share 38% amino acid sequence identity. The sequence identity between Prod1 and TC00268 is only 24%, similar to the 22% identity between human CD59 and Prod1. Furthermore, all sequences in the CD59 clade have the five canonical TFP disulfides, while Prod1 has only four. TC00268 was found in the *Ambystoma* expressed sequence tag (EST) database [32], which also includes an eight-cysteine *A. tigrinum* sequence (TC06378) that was consistently recovered in a clade with Prod1. TC06378 and Prod1 show 56% amino acid sequence identity (Figure S7). These observations strongly suggest that TC06378 corresponds to be the *Ambystoma tigrinum* ortholog of Prod1, and that TC00268 is *Ambystoma tigrinum* CD59. Importantly no mammalian (nor other amphibian) sequence was found to cluster with Prod1 and TC06378.

### Concluding remarks

The establishment of a confident molecular phylogenetic tree of the TFP superfamily is not straightforward due the small size of the domain, mutational saturation and the abundance of insertions and deletions. To overcome this difficulty, we constructed a phylogeny of the available TFP 3D structures using structural similarity scores. This allowed us to confidently place the structure of Prod1 close to the TFP domains of other cell-surface receptors and away from the snake toxins. In order to examine the kinship of Prod1 in more detail we computed a sequence-based phylogeny using a structure-based sequence alignment. The consistent outcome of various phylogenetic methods based upon this alignment shows that Prod1 arises in a clade comprising only non-mammalian proteins. It is possible, due to the relatively low resolution of the trees, that the relationship between Prod1 and any mammalian orthologs is currently beyond the detection limit. However if the pattern of the present analysis holds up in the face of ever-expanding sequence depositions, then our results have the important implication that the coding of proximodistal identity in adult vertebrate limb regeneration via Prod1 is specific to Salamandroidea. Moreover we have shown that Prod1 is not the functional homologue of mammalian CD59, as was previously supposed [3].

The conclusions that we have derived from sequence-structure bioinformatic analysis of Prod1 and its relationship to the TFP superfamily are necessarily limited by the absence of the complete sequence of a urodele genome. Our assessment that Prod1 is not found in mammals would have to be corroborated by the analysis of the relevant syntenic regions. Unfortunately, such corroboration would be confounded by the litany of mechanisms that allow for rearrangement of chromosomal DNA over evolutionary time. Arguably the best way to test functional orthology is by genetic or biochemical tests of Prod1 and candidate Prod1 orthologs in cells of relevant origin, a goal towards which we and our colleagues are expending significant effort.

Recently a protein that appears to interact with Prod1 was identified in *N. viridescens* [33]. Epithelial and neuroepithelial cell-derived protein, nAG, displays sequence homology to a family of proteins with a thioredoxin fold known as anterior gradient (AG) proteins, and by itself has dramatic impact upon the regeneration of an amputated newt limb. For example, electroporation of a nAG expression construct into an experimentally denervated limb blastema stimulates cellular proliferation and rescues much of the development of the regenerate that is otherwise arrested in the absence of the nerve. The connection between Prod1 and nAG would appear to bring together the aspects of PD identity and the nerve-dependence of newt limb regeneration. Whilst the basis for the proposed interaction of nAG with Prod1 is not yet understood at the structural level, it is relevant here to note that the molecular phylogeny reported in [33] indicates that despite of its homology to the AG protein from other species, nAG forms a separate clade only with other non-mammalian proteins. The discovery of the roles played by Prod1 and nAG in newt limb regeneration is providing exciting new avenues to further unravel the underlying cellular and molecular mechanisms. The data reported herein provide, not only a high resolution 3D structure of Prod1 that provide a substrate for examination of the potential interaction with nAG and can guide investigation of structure-activity relationships, but also new insight in the likely absence of a directly orthologous system in mammals. The latter conclusion is of interest in the context of understanding regeneration as an evolutionary variable [7] and the potential transferability of concepts surrounding amphibian regeneration, including the properties of the blastema in particular, to applications in human medicine [34].

## Materials and Methods

### Sample preparation

The DNA sequence encoding residues 19 to 88 of Prod1 was amplified by PCR from full-length Prod1 [3] and ligated into the pET15b vector (Novagen). Transformed *E. coli* BL21(DE3) Gold (Stratagene) cells were grown in PG medium [35] containing 50 µg/ml carbenicillin, prepared using ^15^NH4Cl and/or ^13^C glucose for isotope-labeled samples. Protein expression at 37°C (300 rpm) was induced by addition of IPTG to 1 mM and continued for 12 hours. Cells were harvested and lysed by sonication, the recovered pellet resuspended in 0.1 M potassium phosphate, 10 mM Tris-HCl, 6 M guanidinium chloride (GdmCl) pH 8.0. The supernatant was purified by gravity-flow IMAC and the eluant concentrated to ∼5 ml. Solid DTT was added to 0.1 M, the pH adjusted to pH 8.5, and the solution was incubated for 2 hrs at RT. Size-exclusion chromatography was performed on a Superdex 75 column (GE Healthcare) equilibrated with a pH 4.5 buffer of 0.1 M potassium phosphate, 10 mM Tris-HCl and 6 M GdmCl. Fractions corresponding to the protein monomer were pooled and concentrated. In vitro folding was performed at room temperature by rapid dilution into 0.1 M Tris-HCl pH 9 buffer with 5 mM cysteine and 0.5 mM cystine. The mixture was gently stirred for 48 hours, concentrated and loaded onto Superdex 75 equilibrated in 0.05 M potassium phosphate buffer pH 6.0, 0.2 M NaCl, 1 mM EDTA, 0.1% NaN3. Eluate fractions containing the Prod1 monomer were pooled and concentrated. All concentration steps were carried out by centrifugal ultrafiltration (Vivaspin 20, Vivascience).

### NMR spectroscopy and structure calculation

NMR spectra were acquired at 298 K at 500, 600 or 800 MHz. Sequence-specific and side chain resonance assignments were obtained using standard nD triple resonance methods. All spectra were processed using NMRpipe [36] and analyzed using ANSIG v3.3 [37]. Chemical shifts were indirectly referenced to 2,2-dimethyl-2-silane-pentane-5-sulphonate. Data were deposited in BioMagResBank with code 15477. Interproton distance restraints were derived from ^15^N- and ^13^C-edited NOESY-HSQC spectra. Cross peaks were assigned manually, grouped into four categories according to their relative peak intensities which correspond to interproton distance restraint limits of 1.8–2.5 Å (strong), 1.8–3.0 Å (medium), 1.8–3.5 Å (weak) and 1.8–5.0 Å (very weak). For NOEs involving methyl groups, 0.5 Å was added to the distance upper limit. Only NOEs deriving from unambiguously assigned cross peaks were used in the calculations. Backbone ϕ and ψ torsion angle restraints were derived from the pattern of ^1^Hα, ^13^Cα, ^13^Cβ, ^13^C' and ^15^NH chemical shifts according to the program TALOS [38]. Hydrogen bond restraints for amide protons applied in the final structure calculation were derived from assessment of the regular secondary structure elements of conformers in the early rounds of structure calculations. Conformers were calculated from the experimental restraints using CNS [39] with the PARALLHDGv5.3 parameter set [40], [41] and PROLSQ non-bonded energy function. In order to improve the quality of the final structures, a final step of restrained MD with inclusion of explicit water was used. The final ensemble consists of the 20 lowest energy conformers, deposited in the Protein Data Bank with accession code 2JVE. Structural quality of was assessed with PROCHECK [42], [43] and WHATIF [44].

### Structure alignment and structure phylogenetic analysis

Solved 3D structures of TFP domains were located using the advanced search of the PDB website, searching for structures with the SCOP fold snake toxin-like (57301) or CATH topology CD59 (2.3.60). Fifty-nine none-identical sequences are retrieved, one of them (1QM7) is a chimeric protein, and was removed. To find non-annotated entries the structure of Prod1 was submitted to the FATCAT server to search against the PDB of Nov. 25, 2008, the only new structure found was the muscarinic toxin MT7 (PDB code: 2VLW). The structure of the ectodomain of the type II BMP receptor (2HLQ) and uPAR (2FD6) were not retrieved by these methods, but were also included in the analysis, in the case of uPAR as three independent domains. All the atomic coordinates were obtained from the PDB and trimmed to comprise only the nominal TFP domain. We computed pairwise structure superpositions to obtain a series of similarity scores using default parameters with ASH [45], DaliLite 2.4.5 [46], FATCAT [17] and PPM [23]. To obtain the distance score between structures A and B (D_AB_) we used the formula D_AB_ = S_AA_+S_BB_−2*S_AB_, where S is the similarity score, this guarantees that the self-distance is zero, and all distances are positive. Phylip-like distance matrices were created with in-house Perl scripts. Neighbornet networks were calculated from the matrices by SplitsTree4 [27] and phylogenetic trees by BIONJ [24].

### Sequence alignment and sequence phylogenetic analyses

Vertebrate sequences in UniprotKB were mined using the pattern search tool at the PIR website (http://pir.georgetown.edu/pirwww/search/pattern.shtml) and also ScanProsite at the ExPASy server. The sequence pattern used was C-x(5,30)-C-x(2,10)-C-x(10,30)-C-x(2,20)-C-x(5,30)-C-C-x(4)-C-N; the matching sequences, as well as the TFP sequences found in the venom gland of the Bushmaster snake [47], were pooled with those of the PFAM family PF00021, and then aligned with MUSCLE [48]. The TFP domain could then be extracted from the rest of the sequence. Sequences with more than one TFP domain were split, and incomplete, false positives and highly similar (≥95%) sequences were removed. At this point, the sequences of SMART [49] family SM00134 were incorporated into the dataset. Highly similar (≥95% identical), and incomplete sequences were discarded. Due to the high computational cost of phylogenetic methods, we removed the snake toxins, the TFP domains found in Ser/Thr kinases, the sequences of uPARs and BMP and activin membrane-bound inhibitors (BAMBI), as well as the sequences from non-Craniata species. The protein sequences of Prod1 and human CD59 were used as queries to mine related sequences in the *Ambystoma* EST database [32] and the 18 matching sequences were incorporated into dataset. A 3D structure-based sequence alignment was computed for the proteins in the receptor-like cluster (see results) using the program MUSTANG [50]. The resulting sequence alignment was used as constraint to compute a structure-based alignment of the dataset using MAFFT [51] with L-INS-I –seed settings. The redundancy cutoff of the final alignment was 90% and consisted of 196 sequences.

The most appropriate model of amino acid replacement was computed with the program Prottest 1.4 [52] using four gamma rate categories, and was determined to be WAG [53] with gamma-distributed rates and a proportion of invariant sites (WAG+4G+I). The estimated gamma shape parameter (alpha) was 1.72 and the value of the proportion of invariant sites 0.04. Molecular phylogeny was estimated by maximum-likelihood with PhyML 2.4.4 [54] and by Bayesian inference using MrBayes 3.1.2 [55]. PhyML was run with 1000 bootstrap resampled datasets using the values of alpha and the proportion of invariable sites obtained with Prottest. A majority rule consensus tree was then calculated with Consense of the Phylip suite [56]. MrBayes was run with default parameters sampling every 200 generations for ten million generations after which the log-likelihood values had converged, as judged by the shape of the log probability plot. The final average standard deviation of split frequencies at the end of the run was 0.036. The posterior probabilities and the majority rule consensus tree were calculated after removing the first 12500 trees. Maximum-likelihood pairwise distance matrices using WAG+4G+I were calculated with Treepuzzle 5.2 [57] and a neighbornet network was computed using SplitsTree4. Sequences were visualized and manipulated using Jalview [58] and ClustalX [59]. Tree files were analyzed, viewed and prepared for publication using Dendroscope [60].

## Supporting Information

Figure S1 ^1^H-^15^N Heteronuclear single-quantum coherence (HSQC) spectrum of Prod1 at 298 K and pH 6.0.
(0.27 MB TIF)

Figure S2 Neighbor-net network of TFP 3D structures calculated using the matrix of pairwise distances computed by the phenotypic plasticity method (PPM). The structure-based phylogenetic groupings are circled and highlighted by different colors. The arrows signal the split that separates the snake toxin cluster from the receptor cluster on which Prod1 is located.
(0.95 MB TIF)

Figure S3 3D structure-based cladogram of TFP domains. The trees were computed with BioNJ using a matrix of pairwise distances calculated using DALI similarity scores. PDB codes in bold correspond to the structure depicted in ribbon representation; PDB codes in white correspond to structures whose classification differs from that in the tree computed using PPM scores (Figure 2)
(0.99 MB TIF)

Figure S4 3D structure-based cladogram of TFP domains. The trees were computed with BioNJ using a matrix of pairwise distances calculated using FATCAT similarity scores. PDB codes in bold correspond to the structure depicted in ribbon representation; PDB codes in white correspond to structures whose classification differs from that in the tree computed using PPM scores (Figure 2)
(0.98 MB TIF)

Figure S5 3D structure-based cladogram of TFP domains. The trees were computed with BioNJ using a matrix of pairwise distances calculated using ASH similarity scores. PDB codes in bold correspond to the structure depicted in ribbon representation; PDB codes in white correspond to structures whose classification differs from that in the tree computed using PPM scores (Figure 2)
(0.99 MB TIF)

Figure S6 Neighbor-net network of representative TFP sequences calculated using maximum-likelihood distances estimated using the WAG+4G+I model. The sequence-based phylogenetic groupings are labeled, roman numerals refer to groups that do not have any previously-characterised members. For the description of the sequences in each grouping see Tables S2 and S3.
(4.19 MB TIF)

Figure S7 Multiple alignment of the sequences of Prod1 from eastern newt (*Notophthalmus viridescens*) and from tiger salamander (*Ambystoma tigrinum*) and selected CD59 orthologs. Glycines are colored in orange, prolines in yellow and cysteines in pink; other positions are colored according to conservation of chemical properties: hydrophobic in blue, aromatic in cyan, polar negative in purple, polar positive in red, and polar neutral in green.
(2.85 MB TIF)

Table S1 Pairwise structure distances for the representative set of TFP domain 3D structures used in the structure-based phylogenetic analysis. The distance (D) between any given structures A and B was calculated using the formula D_AB_ = S_AA_+S_BB_−2*S_AB_, where S is the similarity score calculated by the phenotypic plasticity method (PPM).
(0.04 MB XLS)

Table S2 Groupings, accession numbers and descriptions of the sequences found in the phylogenetic tree depicted in Figure 4. Families are arranged in alphabetical order. Individual sequences within a family are arranged corresponding to a clockwise readout of the tree branches in Figure 4.
(0.04 MB XLS)

Table S3 Groupings, accession numbers, and descriptions of the sequences found in the phylogenetic tree depicted in Figure 5. Families are arranged in alphabetical order. Individual sequences within a family are arranged corresponding to a clockwise readout of the tree branches in Figure 5.
(0.04 MB XLS)
