# Supplementary figures and images for: Solution Structure and Phylogenetics of Prod1, a Member of the Three-Finger Protein Superfamily Implicated in Salamander Limb Regeneration
Source: PLoS One. 2009 Sep 22;4(9):e7123. doi: 10.1371/journal.pone.0007123 (PMC2740830; doi:10.1371/journal.pone.0007123)

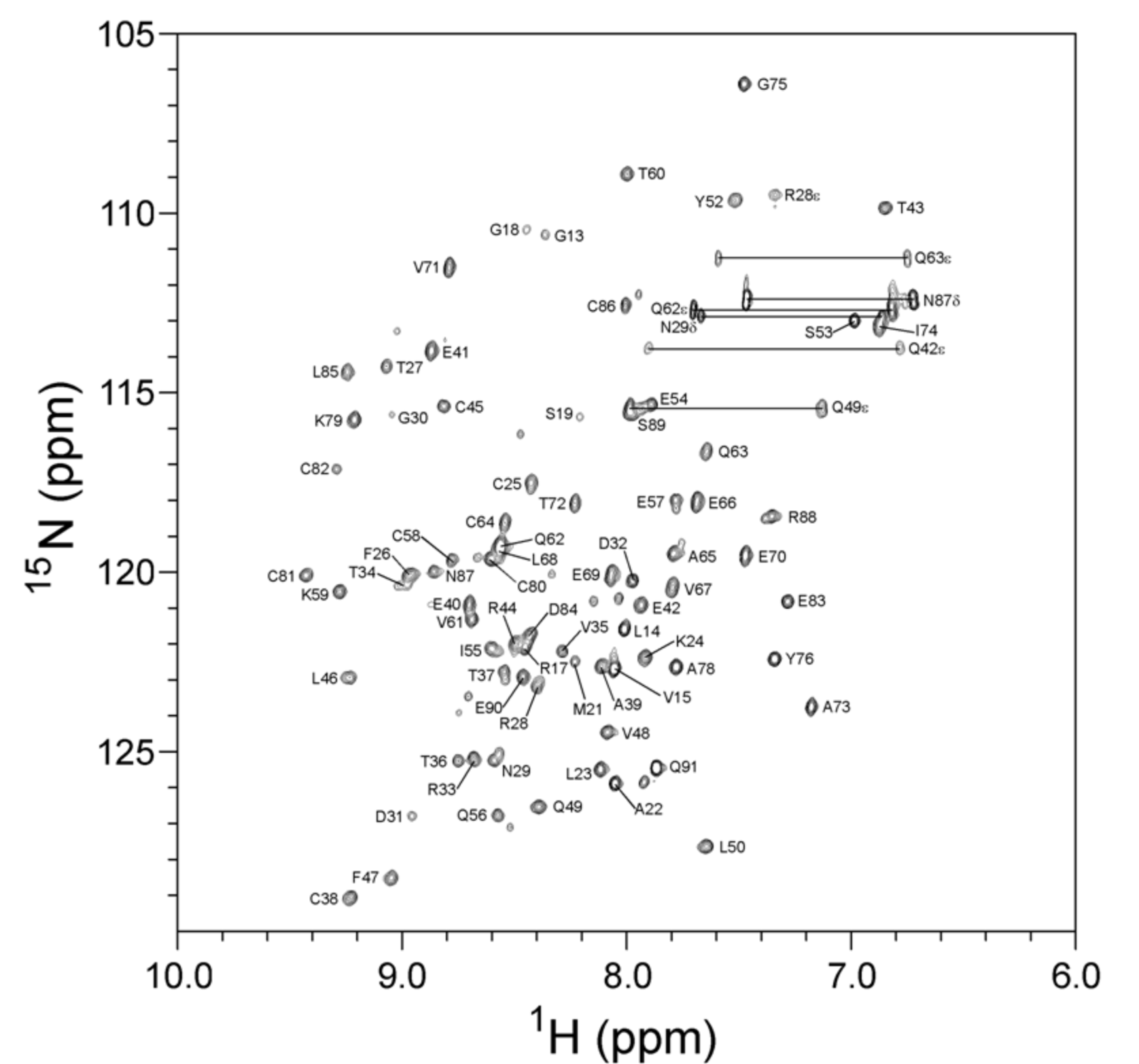

Supplement: Figure S1 — 1H-15N Heteronuclear single-quantum coherence (HSQC) spectrum of Prod1 at 298 K and pH 6.0. (0.27 MB TIF) [file pone.0007123.s001.tif]

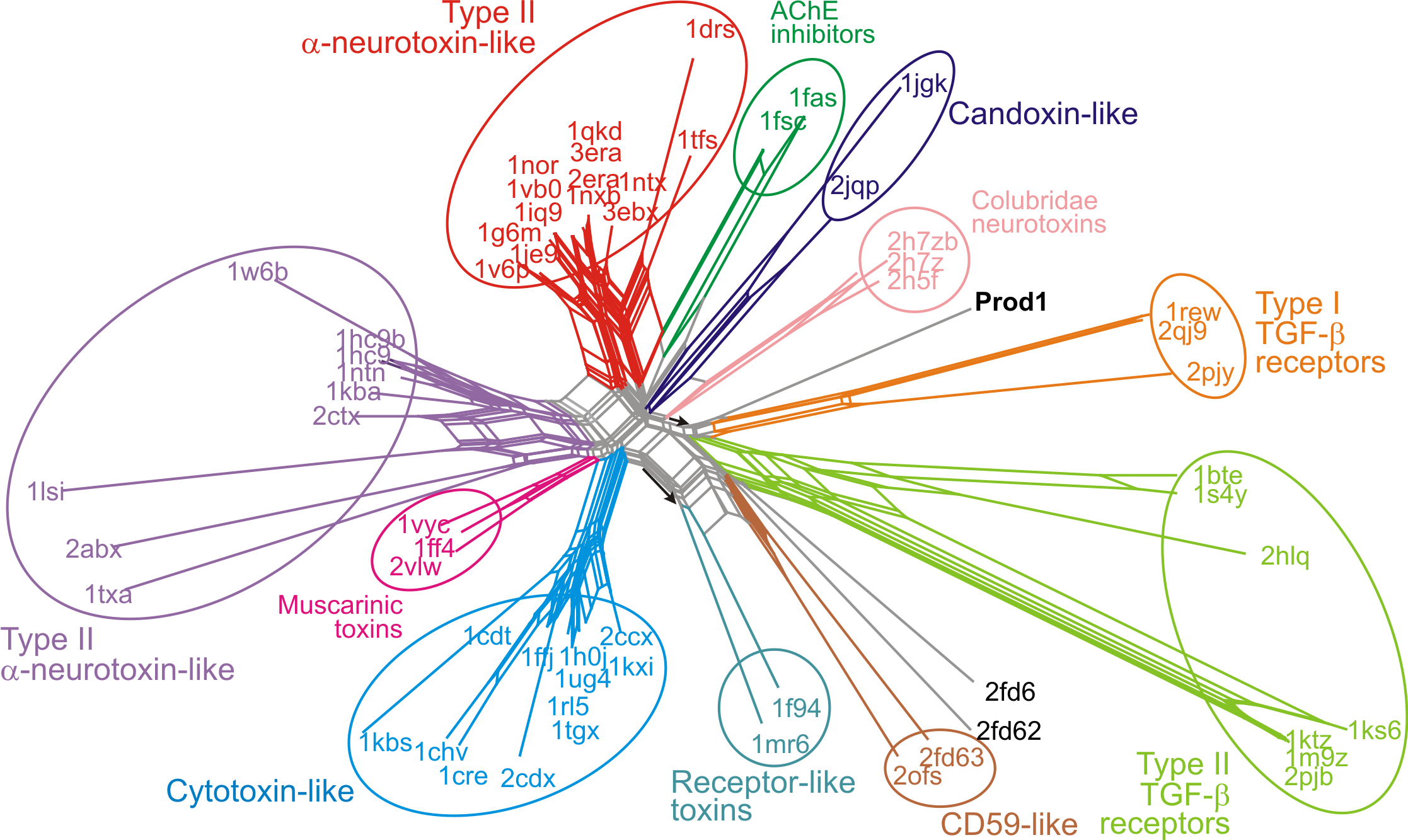

Supplement: Figure S2 — Neighbor-net network of TFP 3D structures calculated using the matrix of pairwise distances computed by the phenotypic plasticity method (PPM). The structure-based phylogenetic groupings are circled and highlighted by different colors. The arrows signal the split that separates the snake toxin cluster from the receptor cluster on which Prod1 is located. (0.95 MB TIF) [file pone.0007123.s002.tif]

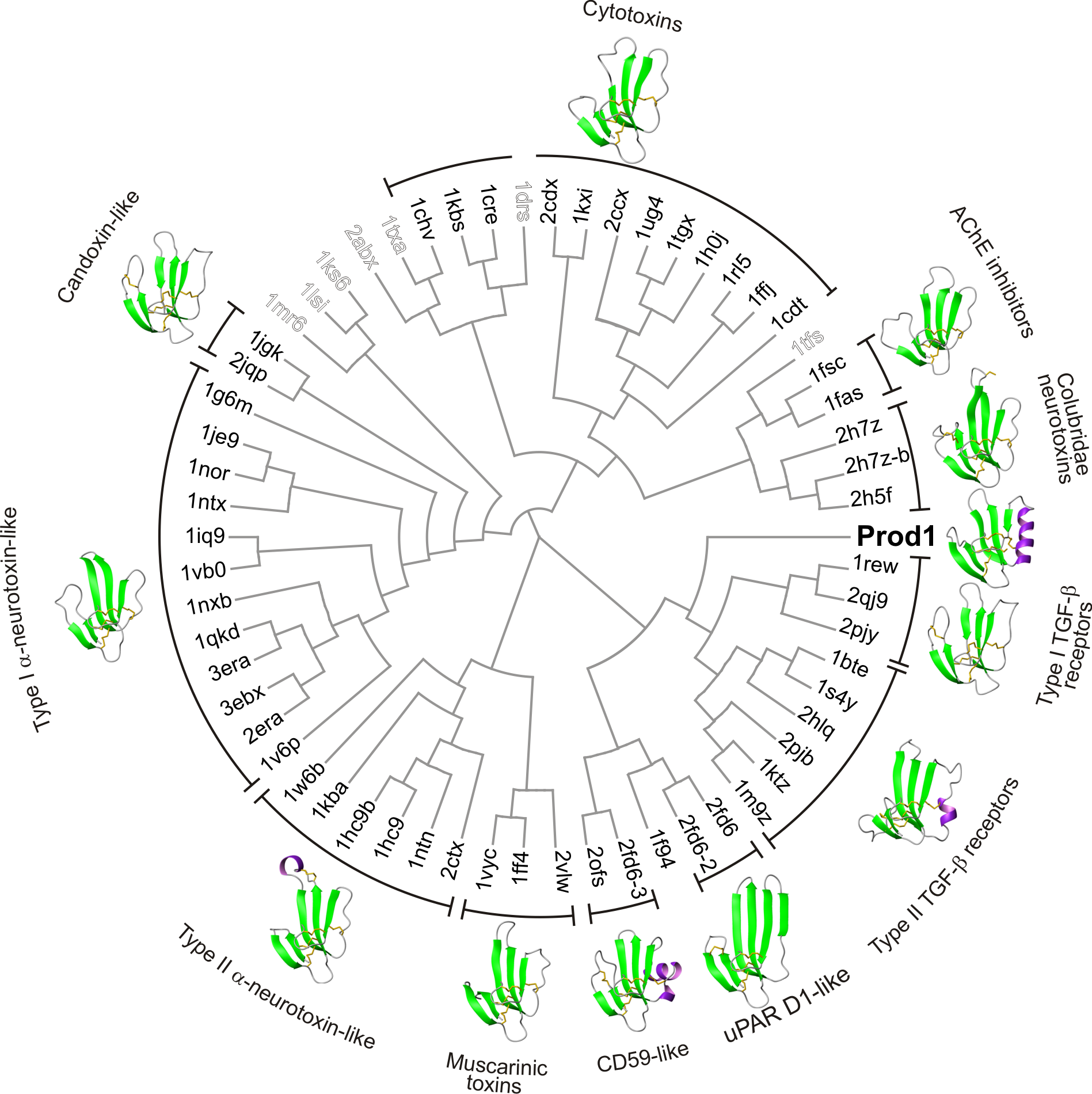

Supplement: Figure S3 — 3D structure-based cladogram of TFP domains. The trees were computed with BioNJ using a matrix of pairwise distances calculated using DALI similarity scores. PDB codes in bold correspond to the structure depicted in ribbon representation; PDB codes in white correspond to structures whose classification differs from that in the tree computed using PPM scores (Figure 2) (0.99 MB TIF) [file pone.0007123.s003.tif]

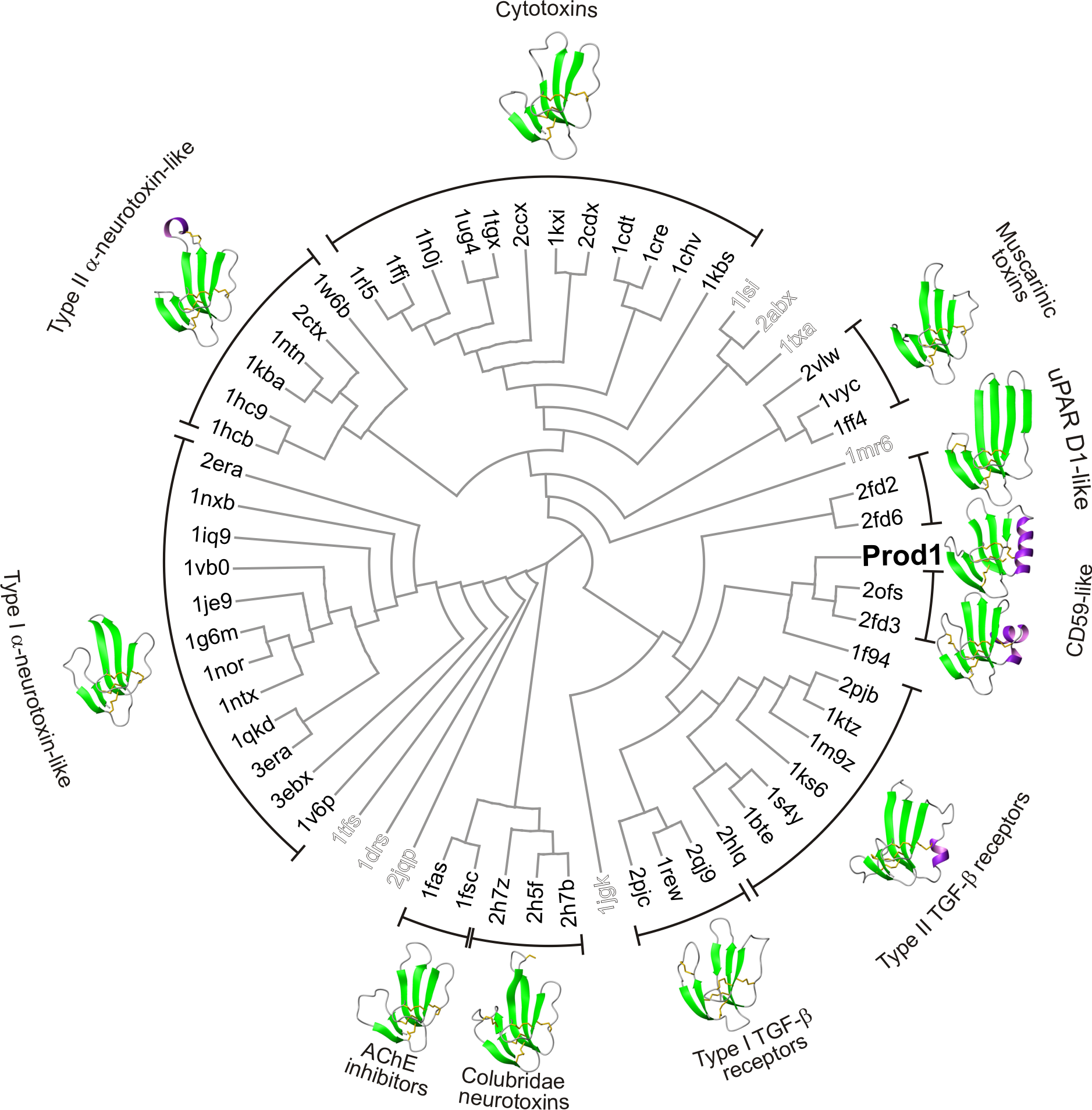

Supplement: Figure S4 — 3D structure-based cladogram of TFP domains. The trees were computed with BioNJ using a matrix of pairwise distances calculated using FATCAT similarity scores. PDB codes in bold correspond to the structure depicted in ribbon representation; PDB codes in white correspond to structures whose classification differs from that in the tree computed using PPM scores (Figure 2) (0.98 MB TIF) [file pone.0007123.s004.tif]

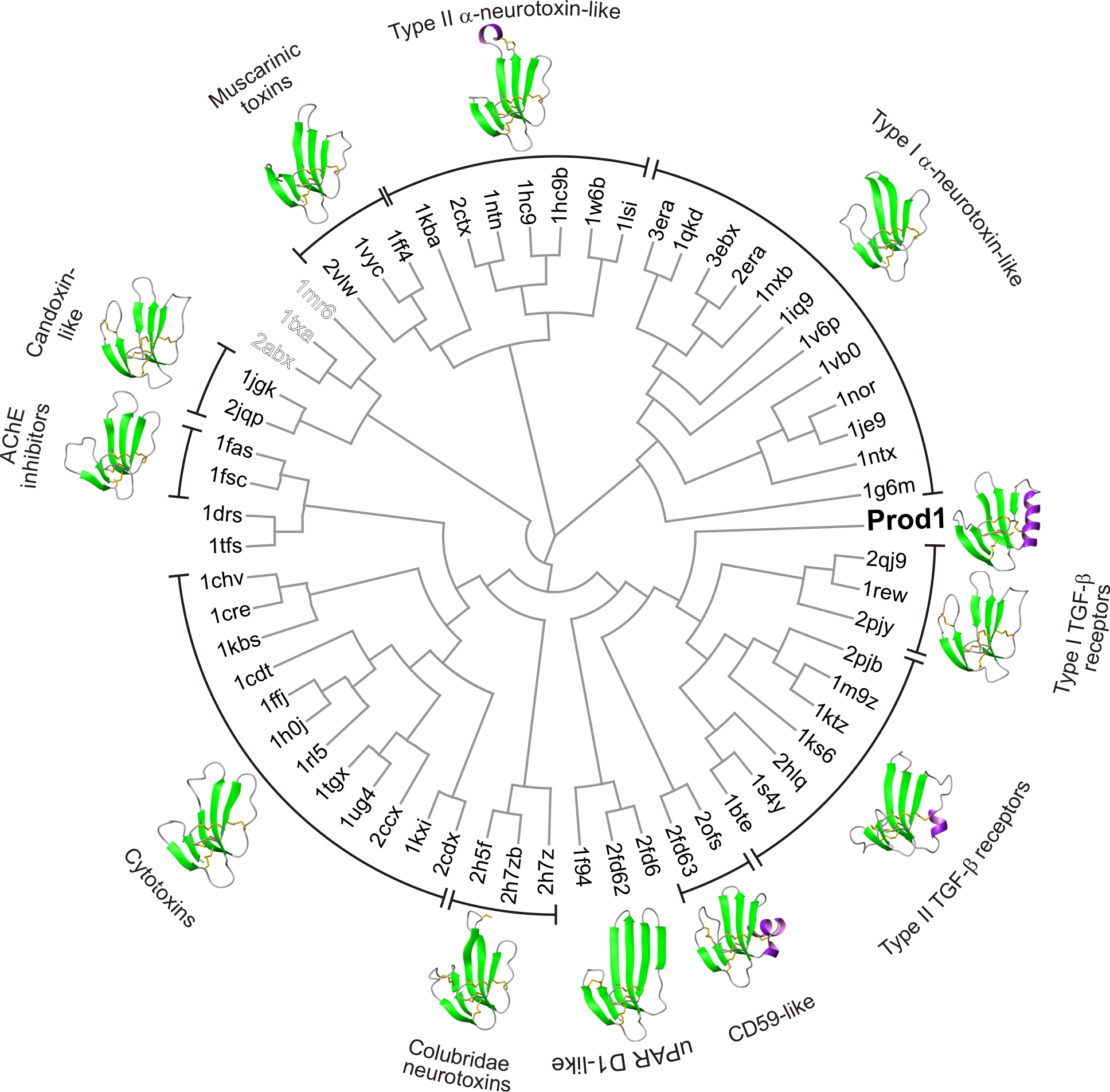

Supplement: Figure S5 — 3D structure-based cladogram of TFP domains. The trees were computed with BioNJ using a matrix of pairwise distances calculated using ASH similarity scores. PDB codes in bold correspond to the structure depicted in ribbon representation; PDB codes in white correspond to structures whose classification differs from that in the tree computed using PPM scores (Figure 2) (0.99 MB TIF) [file pone.0007123.s005.tif]

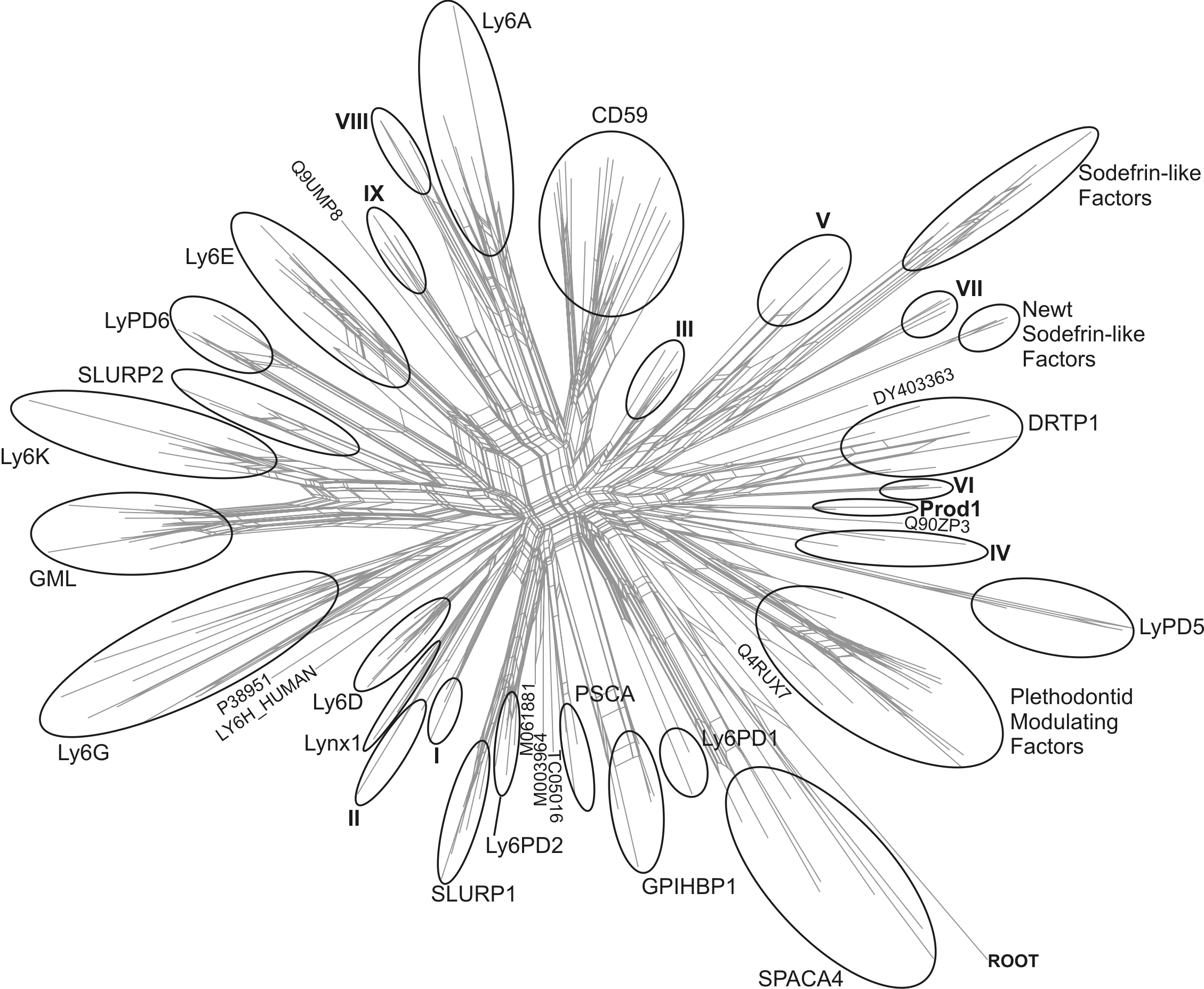

Supplement: Figure S6 — Neighbor-net network of representative TFP sequences calculated using maximum-likelihood distances estimated using the WAG+4G+I model. The sequence-based phylogenetic groupings are labeled, roman numerals refer to groups that do not have any previously-characterised members. For the description of the sequences in each grouping see Tables S2 and S3. (4.19 MB TIF) [file pone.0007123.s006.tif]

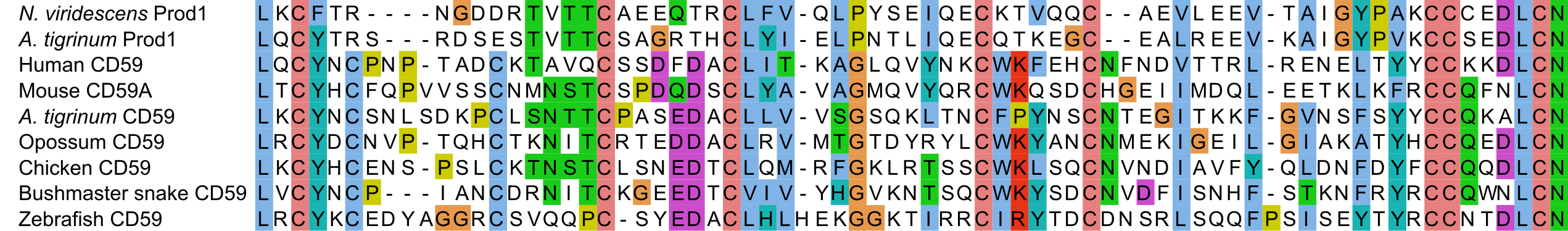

Supplement: Figure S7 — Multiple alignment of the sequences of Prod1 from eastern newt (Notophthalmus viridescens) and from tiger salamander (Ambystoma tigrinum) and selected CD59 orthologs. Glycines are colored in orange, prolines in yellow and cysteines in pink; other positions are colored according to conservation of chemical properties: hydrophobic in blue, aromatic in cyan, polar negative in purple, polar positive in red, and polar neutral in green. (2.85 MB TIF) [file pone.0007123.s007.tif]
